# Physicochemical, Antioxidant, and Anti-Inflammatory Properties of Rapeseed Lecithin Liposomes Loading a Chia (*Salvia hispanica* L.) Seed Extract

**DOI:** 10.3390/antiox10050693

**Published:** 2021-04-28

**Authors:** Ailén Alemán, Selene Pérez-García, Pilar Fernández de Palencia, María Pilar Montero, María del Carmen Gómez-Guillén

**Affiliations:** Institute of Food Science, Technology and Nutrition (ICTAN-CSIC), C/José Antonio Novais, 10, 28040 Madrid, Spain; ailen@ictan.csic.es (A.A.); s.perez@ictan.csic.es (S.P.-G.); pfpalencia@ictan.csic.es (P.F.d.P.); mpmontero@ictan.csic.es (M.P.M.)

**Keywords:** rapeseed lecithin, liposomes, chia seeds, nanoencapsulation, stability, anti-inflammatory

## Abstract

Vegetal waste materials were used to produce liposomes with both antioxidant and anti-inflammatory properties. Differences in the chemical composition of rapeseed lecithin (LEC) and a partially purified phospholipid fraction (PPL) were studied in terms of fatty acids (neutral lipids, free fatty acids, and phospholipids), sterols, tocopherols, and amino acid composition. Neutral lipids, campesterol, β-sitosterol, and γ-tocopherol were the most depleted compounds in PPL. Qualitative differences between LEC and PPL were revealed by infrared spectroscopy and differential scanning calorimetry. An ethanol/water antioxidant extract from chia seeds (ChE), with a high content in rosmarinic acid and rosmarinic acid 3-O-glucoside, along with other minor phenolic acids determined by HPLC-MS, was encapsulated in liposomes made of LEC (L-LEC) and PPL (L-PPL) with an entrapment efficiency of 61.3% and 69.3%, respectively. L-PPL suspensions showed smaller particle size and lower ζ potential than their L-LEC counterparts, along with noticeable particle destabilization after 7 days of storage. Antioxidant properties were greater in L-LEC than in L-PPL suspensions. L-LEC, ChE, and lecithin empty liposomes (L-E) showed no cytotoxic effect in either Caco-2 or THP-1 cells and induced downregulation of the inflammation response.

## 1. Introduction

There is growing demand for natural food ingredients rich in bioactive compounds. In particular, polyunsaturated fatty acids (PUFA), phytosterols, and phenolic compounds have been related to reduced risk of cardiovascular and neurodegenerative diseases, diabetes, and cancer owing to their anti-inflammatory and antioxidant properties [1]. Residues from vegetable oil processing may constitute a sustainable source of such compounds, which could be used for the production of novel functional ingredients with high added value and no legal restrictions [2].

Lecithin is an abundant residue generated during the degumming step in the vegetable oil refining process. In addition to well-known technological uses as a food additive (E-322, natural emulsifier), lecithin may also be considered a functional food ingredient owing to its phospholipid composition rich in polyunsaturated fatty acids, and especially phosphatidylcholine, which has been shown to improve brain function [3]. Interest in non-soy lecithin has increased in view of concerns regarding GMO in soybeans and possible food allergic reactions. Rapeseed (*Brassica napus*), the second most important oilseed crop after soy, has been investigated as an alternative, naturally occurring phospholipid source to produce liposomes for the encapsulation of bioactive compounds [4,5,6]. Liposomes, which are spherical vesicles formed by one or more phospholipid bilayers, can be used to enrich functional foods with healthy and bioactive compounds, both hydrophilic and hydrophobic, including antioxidants, vitamins, minerals, peptides, and enzymes [2]. Furthermore, the liposomal encapsulation improves both physical and chemical stability of the bioactive compounds during food processing, prevents their possible loss of activity by interactions with other food constituents, and favors their oral bioavailability [2]. The main drawback in the use of highly unsaturated vegetal phospholipids is their strong susceptibility to lipid oxidation [7], which may contribute to rancid off-flavor development in food. In the context of liposomal preparations, the predominance of unsaturated phospholipids typically produces liposomes with reduced thermal stability [8] and increased membrane fluidity, which may become more rigid due to easy oxidation [9]. In addition to a high PUFA content, rapeseed lecithin has also been reported to contain significant amounts of sterols, carotenoids, and tocopherols with antioxidant properties [10]. As reported for partially purified soy phosphatidylcholine liposomes, residual amounts of impurities, including antioxidants, would generally be admissible to obtain derived liposomes with suitable structural properties for food applications, and moreover, provide endogenous protection against oxidation [11]. Furthermore, the loading of liposomes with antioxidant polyphenolic compounds has also been reported to reduce the formation of undesirable oxidation products [12].

Seeds from chia (*Salvia hispanica* L.), an herbaceous plant of the *Lamiaceae* family, represent an abundant source of omega-3-linolenic acid-rich oil, along with high biological value proteins, dietary fiber, and minerals [13]. The oil extraction process from chia seeds produces a residual cake rich in phenolic compounds such as chlorogenic acid, rosmarinic acid, caffeic acid, salicylic acid, kaempferol, rutin, myricetin, and quercetin, with a potent antioxidant activity and protective capacity against genotoxic and mutagenic activities in human leukocytes [14,15,16]. Clinical effects of chia seeds intake on human health have been exhaustively reviewed, the most significant being those related to the lowering of diastolic blood pressure, postprandial blood glucose levels, and high-density lipoprotein in serum [17].

The aim of the present work was to study the effect of chemical composition of both rapeseed lecithin and a derived partially purified phospholipid fraction on the physicochemical, antioxidant, and anti-inflammatory properties of the resulting liposomes loaded with an active extract obtained from a de-oiled residual cake of chia seeds.

## 2. Materials and Methods

### 2.1. Materials

Commercial rapeseed lecithin (LEC) was kindly provided by Bunge (R-1000, BungeMaxx, Rotterdam, The Netherlands). Chia (*Salvia hispanica* L.) seeds originally from Perú (Grainway, Pedon S.P.A., Colceresa, VI, Italy) were acquired in a local market. The standards used were all from Sigma-Aldrich, namely α-tocopherol (Ref. 47783), δ-tocopherol (Ref. 47784), γ-tocopherol (Ref. 47785), β-sitosterol (Ref. 43623), stigmasterol (Ref. S2424), and campesterol (Ref. C5157). All other reagents were of analytical grade.

#### Obtaining of Chia Seed Extract

Chia seeds were treated with liquid nitrogen and crushed in an Osterizer blender to a homogeneous powder. The crushed seeds were then treated for 15 min at 90 °C for enzyme inactivation. Afterward, they were vacuum packed (model A300/52 multivac, Germany) and stored at −20 °C until use. The seed powder was de-oiled with hexane, at a ratio of 1:1.5 (seed powder:hexane, *w*/*v*) by stirring for 30 min. The upper phase was separated by decantation and filtered, constituting the oil fraction. This hexane-washing process was repeated four more times to extract as much oil as possible. The resulting solid fraction (cake) was dried in an oven at 45 °C for 24 h. The chia seed extract was obtained by homogenizing 50 g of de-oiled cake with 100 mL of ethanol:water (1:1). The suspension was adjusted to pH 2, homogenized in an IKA Ultraturrax blender (Staufen, Germany), and then sonicated (2 cycles of 2 min with a 1-min rest) using an ultrasound probe (model Q700, Qsonica sonicators, Newtown, CT, USA) at 100% amplitude. The homogenate was centrifuged at 12,000× *g* for 10 min at 5 °C. The supernatant was rotaevaporated to remove ethanol, frozen at −80 °C and freeze-dried. The analysis of phenolic composition was performed by reverse-phase high-performance liquid chromatography (RP-HPLC) on a C18 analytical column, following the procedure described in Alemán et al. [18]. Rosmarinic acid and rosmarinic acid-3-O-glucoside were quantified using a calibration curve of the corresponding standard compounds.

### 2.2. Identification of Phenolic Compounds by HPLC-ESI-QTOF Analysis

Phenolic compounds present in the chia extract were characterized by HPLC-ESI-QTOF MS. The separation of polyphenols was carried out at 25 °C in an Agilent 1200 MSD 6530A-QTOF accurate mass liquid chromatograph (Agilent Technologies, Waldbronn, Germany), equipped with a diode array detector (DAD) and a thermostatic injector, and coupled to a mass spectrometer QTOF (Agilent G6530A Accurate Mass Q-TOF LC-MS) equipped with an ESI polarization source (negative polarity) with JetStream technology. A Tracer Excell 120 ODS-A column was used for separation. Gradient elution was performed with a binary system formed by ultrapure water with 0.1% (*v*/*v*) acetic acid (solvent A) and methanol:acetonitrile:water (50:25:25) with 0.1% (*v*/*v*) acetic acid (solvent B) at a flow rate of 0.6 mL/min. The system was run with the following gradient program: 0–5 min at 10% B, 5–45 min from 10 to 100% B, 45–50 min at 100% B, 50–55 min from 100 to 10% B, 55–60 min at 10% B. The spectra were recorded at wavelengths of 253, 280, 368, and 520 nm. Data were acquired in negative ion mode, with an m/z range of 100–1200, using a source temperature of 325 °C and a gas flow of 12 L/min. The auto MS/MS acquisition was used. The spectra obtained were analyzed with the MassHunter Qualitative Analysis B.07.00 software. The phenolic compounds were identified according to their retention times, according to the molecular formula proposed by the software and by comparing the experimental mass with the exact mass. Ion fragmentation was also compared with the fragmentation patterns described for phenolic compounds in public databases and in bibliographic references to support the identification. Quantification of rosmarinic acid and rosmarinic acid 3-O-glucoside was carried out by using the standard curve of rosmarinic acid, considering the chromatogram peak areas from the diode array detector (DAD) at 253 nm.

### 2.3. Preparation of Liposomes

A partially purified phospholipid-rich fraction (PPL) obtained from commercial rapeseed lecithin (LEC) was prepared following the method of Taladrid et al. [11] by applying five washes (30 min) with acetone. Liposomes from PPL (L-PPL) and LEC (L-LEC), both loaded with the chia seed extract, were prepared by dissolving 1.6 g of freeze-dried chia extract in phosphate buffer at pH 7 and mixing with 5 g of LEC or PLL (L-LEC and L-PPL), according to Marín et al. [2]. Empty liposomes (E-L) without the chia extract were also prepared for comparative purposes.

### 2.4. Chemical Composition of LEC and PPL

The fatty acid composition was determined in LEC and PPL (total fatty acids) and, after fractionation, in each lipid fraction (neutral lipids, NL; free fatty acids, FFA, and phospholipids, PL), following the procedure described by Taladrid et al. [11]. Tocopherols were determined according to Taladrid et al. [11], using liquid chromatography coupled to triple Q mass spectrometry. For sterol determination, GC-FID methodology was used following the method of Du and Ahn [19], using an Agilent 6890A gas chromatograph coupled to an FID (320 °C) detector, with an Agilent HP-5MS column and helium (1.2 mL/min) as carrier gas. The amino acid composition was determined as described by Taladrid et al. [11] using norleucine as standard. The total protein content was calculated from the amino acid composition.

### 2.5. Infrared Spectroscopy (ATR-FTIR)

Both LEC and PPL preparations were further characterized by ATR-FTIR as described previously [11], averaging 32 interferogram scans for each spectrum. Measurements were performed at least in triplicate.

### 2.6. Differential Scanning Calorimetry (DSC)

Thermal analysis of LEC and PPL preparations was performed using a model TA-Q1000 differential scanning calorimeter (DSC) (TA Instruments, New Castle, DE, USA). The instrument was calibrated with high-purity indium (melting enthalpy, 28.44 J/g; melting point, 156.4 °C). Samples of around 8–10 mg were tightly encapsulated in hermetic aluminum pans, using an empty pan as reference. They were scanned under dry nitrogen purge (50 mL/min) from −50 to 90 °C, at a heating rate of 1 °C/min. Endothermic peak temperatures (Tm, °C) and enthalpy (∆H, J/g) were determined in triplicate.

### 2.7. Liposome Hydrodynamic Properties

Particle size (expressed as z-average, nm), polydispersity index, and ζ-potential (mV) were determined using a Zetasizer Nano ZS (Malvern Instruments Ltd., Worcestershire, UK), as described previously (11). Measurements were carried out in triplicate.

To evaluate the pH stability, liposome suspensions were diluted 1:10 at pH 2 (glycine HCL buffer), pH 4 (sodium acetate buffer), and pH 6, pH 7, and pH 8 (sodium phosphate buffer) and then shaken vigorously. The hydrodynamic properties were determined in the Zetasizer Nano, both at day 0 (newly produced liposomes) and after 7 days stored in the refrigerator at 4 °C (day 7).

### 2.8. Entrapment Efficiency

The entrapment efficiency (EE) of the chia extract in L-LEC and L-PPL was determined by measuring the Folin-reactive substances (FRS), following the procedure described by Alemán et al. [18]. The EE was calculated using the following equation:

% EE = encapsulated FRS/total FRS × 100

### 2.9. Antioxidant Properties

ABTS radical scavenging capacity (ABTS assay, Vitamin C eq.), Ferric ion reducing power (FRAP assay, mM Fe^2+^ eq.), and total Folin-reactive substances (FRS, gallic acid equivalent) were determined for empty lecithin liposomes (E-L) and lecithin liposomes loaded with the chia extract (L-LEC), as described in a previous work [18]. The antioxidant properties of the freeze-dried chia extract were also determined.

### 2.10. Cytotoxicity and Anti-Inflammatory Properties

#### 2.10.1. Cell Culture and Cell Viability

Caco-2 and THP-1 cells were obtained from the human cell bank at the Centro de Investigaciones Biológicas Margarita Salas (CSIC) (Madrid, Spain). Caco-2 cells (1 × 10^5^/mL) were maintained in MEM-alpha medium (Invitrogen, Thermo Fisher Scientific, Waltham, MA, USA) supplemented with 10% (*v*/*v*) heat-inactivated fetal bovine serum (Gibco, Thermo Fisher Scientific), streptomycin (100 µg/mL), and penicillin (100 U/mL) (Gibco) at 37 °C in an atmosphere containing 5% CO_2_. The culture medium was replaced every 3 days and a monolayer of 80–90% confluent cells was obtained in 2 or 3 days to mimic small-intestine mature enterocytes.

THP-1 cells (2 × 10^5^/mL) were maintained in RPMI 1640 medium (Gibco Life Technologies) supplemented as described above and were differentiated into macrophages by adding a final concentration of 25 nM phorbol 12-myristate13-acetate (PMA) (Sigma-Aldrich, St. Louis, MO, USA) after incubation for 72 h, as described by Lund et al. [20]. The cells became adherent and PMA was washed using pre-warmed RPMI; the cells were cultured for 24 h in this medium.

Caco-2 cells were seeded at 10^4^ cells/well in 100 μL supplemented culture medium on a 96-well tissue culture plate (Falcon Microtest™, Franklin Lakes, NJ, USA) and incubated to reach a cell confluence of 80–90%, for approximately 2 or 3 days at 37 °C and 5% CO_2_. THP-1 cells (10^5^ cells/well) were treated in the same way and differentiated into macrophages as described above. Then, 10 μL of each sample, tested at two different concentrations (high and low), was added to the wells against the two cellular lines. Final concentrations in complete culture medium were as follows: empty liposomes *L-E* (high = 1000 μg/mL; low = 500 μg/mL), chia extract *ChE* (high = 320 μg/mL; low = 160 μg/mL), and chia extract encapsulated in lecithin liposomes *L-LEC* (high = 1320 μg/mL; low = 660 μg/mL). They were incubated for 18 h, at 37 °C and 5% CO_2_. The supernatant was then removed, and to test cell viability, both human cell types were treated with 110 μL medium Krebs-Henseleit buffer (Sigma-Aldrich) as a control or 100 μL Krebs with 10 μL CCK-8 solution added according to the manufacturer instructions. After that, they were incubated for 1–2 h at 37 °C in a dark place (Cell Counting Kit-8, Sigma-Aldrich, St. Louis, MO, USA). Relative cell viability was determined spectrophotometrically at 450 nm by quantification of the amount of the formazan dye generated by the activity of dehydrogenases in cells, which is directly proportional to the number of living cells.

#### 2.10.2. Immunostimulation

To measure the immune response of the THP-1 cells differentiated into macrophages, 2 × 10^5^ cells/well in 900 μL completed culture medium RPMI were seeded into a 24-well plate. Then, 100 μL of the samples was added at the low final concentrations described above and incubated at 37 °C and 5% CO_2_ for 18 h before or after 4 h of stimulation with LPS (O26:B6, 1 μg/mL). A control (untreated and nonstimulated cells) and a negative control (untreated and stimulated cells) were carried out. After treatment, samples were centrifuged at 1200 rpm for 5 min, and supernatants were recovered and stored at −20 °C until cytokine analysis. The concentration of each cytokine IL-10 and TNF-α released into the supernatants was quantified using an ELISA kit (Diaclone ELISA Kits, Besancon Cedex, France) according to the manufacturer instructions.

### 2.11. Statistical Analysis

Each experiment was repeated three times, and the results were expressed as mean ± SD. Statistical significances were compared between each treated group, and analysis of variance (ANOVA) was performed using SPSS Statistics 26 Software (IBM SPSS Statistics 22 Software, Inc., Chicago, IL, USA). The differences between means were assessed on the basis of confidence intervals using the Tukey tests with significance level set at *p* < 0.05.

## 3. Results and Discussion

### 3.1. Fatty Acid Composition

The fatty acid compositions of rapeseed lecithin (LEC) and the partially purified phospholipid fraction (PPL) are presented in Table 1. The main fatty acids in both samples were, in descending order, oleic acid (C18:1n9c), linoleic acid (C18:2n6c), palmitic acid (C16:0), α-linolenic acid (C18:3n3), vaccinic acid (C18:1n7c), and stearic acid (C18:0). Other fatty acids were present in much lower proportions (<1%). A very similar fatty acid composition has been previously described in phospholipids extracted from rapeseed oil [4]. With the exception of vaccinic acid, all other fatty acids were also found to predominate in soy lecithin, although in varying relative proportions, as noted elsewhere [21]. Linoleic acid was the second most abundant fatty acid in both LEC and PPL. In contrast to rapeseed lecithin, linoleic acid was reported to be the main fatty acid in soy lecithin, representing around 55% of all fatty acids present [7,11]. As with other natural sources, rapeseed lecithin presents a predominance of mono- (MUFA) and poly-unsaturated fatty acids (PUFA). The acetone-washing step led to a slight depletion of MUFA and a relative increase in PUFA and saturated fatty acids (SFA) (*p* < 0.05) (Table 1).

Both LEC and PPL were fractionated into the three main lipid classes, i.e., neutral lipids (NL), free fatty acids (FFA), and phospholipids (PL). LEC was composed of 42.4% PL, which were found in a concentration of up to 88.5% in PPL as a result of consecutive acetone-washing steps. At the same time, there was a significant reduction in the amount of NL and FFA in PPL, contents changing from 52.1% to 8.0% and from 5.5% to 3.6%, respectively. The PL content in LEC was considerably lower than that previously reported for soy lecithin (57.5%) following the same analytical methodology (11), and as in that case, the phospholipid proportion in the resulting PPL preparation was also slightly lower (88.5% in rapeseed vs. 95% in soy).

**Figure 1:**
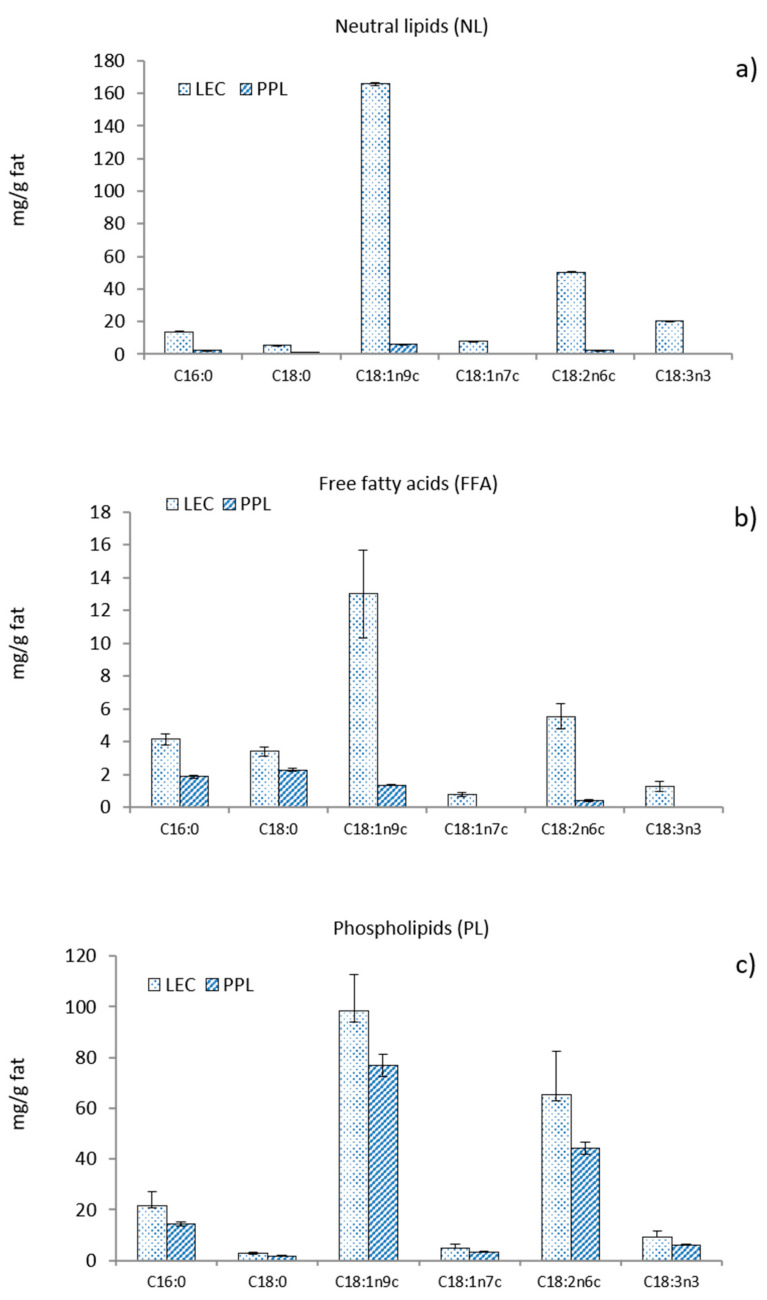
Fatty acid composition of (**a**) neutral lipids, (**b**) free fatty acids, and (**c**) phospholipids from commercial rapeseed lecithin (LEC) and partially purified phospholipid fraction (PPL).

The fatty acid composition of the different lipid fractions is shown in Figure 1. The most abundant fatty acid in LEC was oleic acid, which represents 63% of all fatty acids in NL, 46% in the form of FFA, and 52% in PL. The distribution of linoleic acid among the various fractions in LEC was 19% in NL, 20% in FFA, and 30% in PL. The washing step led to a noticeable removal of both oleic and linoleic acids from NL and also from FFA, inducing a relative increase in the proportion of free saturated fatty acids (C16:0 and C18:0) in PPL. The final concentration of oleic and linoleic acids also decreased in the PL fraction of PPL, although to a lesser extent than in NL and FFA, resulting from the insolubility of phospholipids in acetone.

### 3.2. Sterols, Tocopherols and Amino Acid Composition

Table 1 also shows the concentrations of sterols and tocopherols determined in LEC and PPL. The amounts of campesterol and β-sitosterol, which were substantial in LEC, were considerably depleted in the PPL sample. Stigmasterol and cholesterol were found in trace amounts in LEC, while the latter was absent in PPL. On the other hand, other sterol species such as cycloartenol and a tentatively identified stigmasterol derivative were found in a similar proportion in LEC, but they were not quantified. In particular, the cycloartenol content showed a 5.6-fold decrease (in terms of chromatography area reduction) in the corresponding PPL, while the stigmasterol derivative was not detected in the partially purified preparation. β-Sitosterol, campesterol, and stigmasterol are the major phytosterols in most vegetable oils. Large amounts of stigmasterol have not been reported in rapeseed; instead, brassicasterol was reported to be the third main sterol component [22]. Cholesterol has been detected in the sterol fraction of many vegetable oils as a minor component, 24-dihydrocycloartenol being its most likely precursor [23]. Plant sterols have been widely related to human health benefits, particularly in lowering total plasma cholesterol levels [24]. In addition to other important biological activities, such as antioxidant and anti-inflammatory [25], plant sterols can interact with phospholipid membranes and change their properties in a concentration-dependent manner [26].

Tocopherols (γ, δ and α) were also considerably depleted in PPL as compared with the LEC sample (Table 1), the most abundant being γ-tocopherol. Similar results have been reported in commercial soy lecithin samples compared with their corresponding acetone-washed preparations [7,11]. In contrast to the present work, δ-tocopherol was the most abundant tocopherol species in rapeseed phospholipids extracted from oil degumming [4].

Trace amounts of amino acids were found in both LEC and PPL (Table 1). Most of the amino acid residues tended to concentrate in the PPL preparation, attributed to the acetone-insoluble property of proteins. The predominant amino acids in LEC and PPL were glutamic acid, aspartic acid, and serine, while methionine and cysteine were the least abundant. Based on the amino acid composition, the total protein content increased slightly from 0.215 mg/g in LEC to 0.305 mg/g in PPL. These results were noticeably lower than those previously reported for soy lecithin and partially purified phosphatidylcholine [11], where glutamic acid, aspartic acid, and serine were also among the most prominent amino acids.

### 3.3. Infrared Spectroscopy (ATR-FTIR)

**Figure 2:**
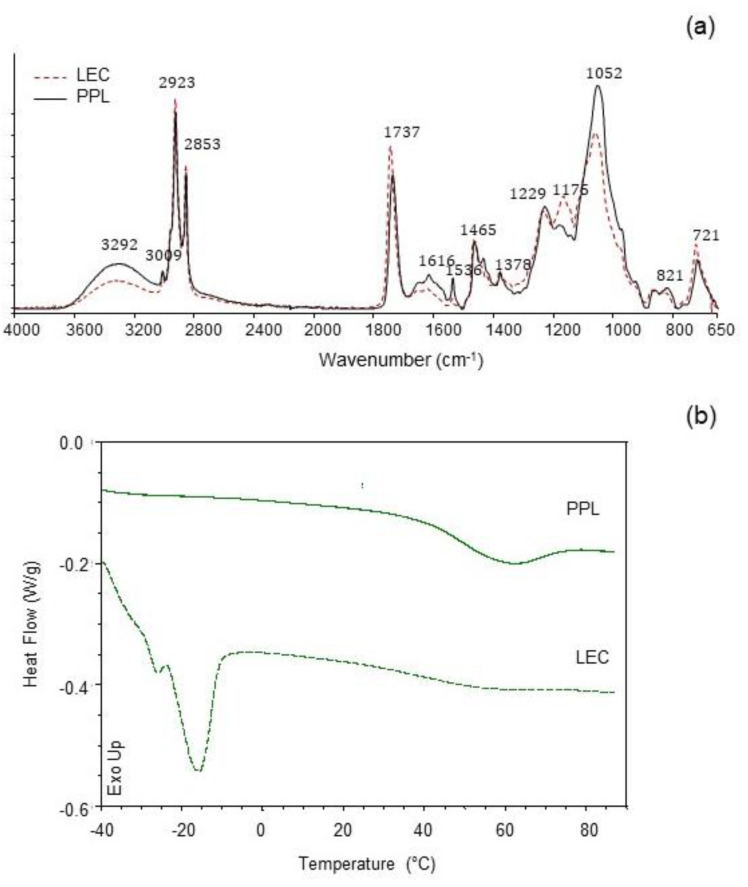
(**a**) Infrared spectra (ATR-FTIR) and (**b**) differential scanning calorimetry (DSC) traces of commercial rapeseed lecithin (LEC) and partially purified phospholipid fraction (PPL).

The IR spectra of LEC and PPL samples are shown in Figure 2a. The PPL was characterized by higher IR band absorption at ≈3290 cm^−1^ (O-H and N-H stretching vibrations), ≈1650 cm^−1^ (C=C stretching vibrations), and ≈1050 cm^−1^ (C-O, C-N and P-O-C stretching vibrations), as compared with the LEC sample. A similar profile was previously reported in a work with soy lecithin and partially purified phosphatidylcholine [11], attributed to the concentration of phospholipids. Furthermore, the reduction in the intensity of the band at ≈1737 cm^−1^ (C=O stretching vibrations) in PPL was also coincidental with a considerable decrease in the total content of neutral lipids (mostly triglycerides). Contributions from phosphate groups in the polar heads of phospholipids were distinguishable at 1229 cm^−1^ (P=O) and 1052 cm^−1^ (POC) [27,28], the latter being noticeably higher in PPL. The peak at 1175 cm^−1^, which could be assigned to stretching vibrations of the CO-O-C group [27,29], was strongly reduced in PPL as compared with the LEC sample, in accordance to the removal of nonphospholipid compounds in PPL. The sharp small peak at 1536 cm^−1^ (stretching vibration of amide II) in PPL, together with the increased IR absorption at 1616 cm^−1^ (stretching vibration of amide I), could possibly be due to a concentration of cerebrosides [30], which have been previously reported in rapeseed lecithin [31]. Such a prominent peak at 1536 cm^−1^ was not observed in partially purified phosphatidylcholine from soy lecithin [11]. Trace amounts of amino acids present in both LEC and PPL could also add some IR absorption assigned to the protein amide bands.

### 3.4. Thermal Properties

Thermal properties were studied to characterize the melting behavior of both LEC and PPL (Figure 2b). The LEC thermal profile showed an intense endothermic event at Tm = −14.8 °C (ΔH = 8.07 J/g), indicative of the main gel-to-liquid-crystalline transition. A small pretransition was observable at −26.09 °C (ΔH = 0.16 J/g). A subzero melting temperature range is typical for lipid compositions with high polyunsaturated fatty acid content [32]. Another slight endothermic transition in LEC was observed at 54.7 °C (ΔH = 0.20 J/g). The PPL preparation, on the other hand, showed the main endothermic transition at Tm = 58.9 °C (ΔH = 4.58 J/g), while slight transitions at subzero temperatures were recorded at −29.4 °C (ΔH = 0.02 J/g) and −15.3 °C (ΔH = 0.003 J/g). Given the differences in the chemical composition of the two preparations, the main subzero endothermic event in LEC could be largely associated with the greater amount of unsaturated bonds with cis configuration found in this sample (C18:1n9c and C18:2n6c), as shown in Figure 1. A similar endothermic transition profile has been reported for the melting of triglycerides from peanut oil [33]. The uneven endothermic transitions at 58.9 °C in PPL and 54.7 °C in LEC could then be attributed in part to the phospholipid fraction, which was predominant in the case of the PPL preparation. As suggested by the FTIR study, the presumptive relative abundance of cerebrosides in PPL could have contributed to the increase in temperature and enthalpy of this second endothermic event [34]. The thermal behavior of LEC reflected a more complex lipid system than PPL, in agreement to the chemical composition.

### 3.5. Liposomal Encapsulation

Both LEC and PPL were assessed for their ability to encapsulate an ethanol/water extract from chia seeds in liposomes. The entrapment efficiency (EE), determined in terms of Folin-reactive substances, was 61.3% in LEC liposomes (L-LEC) and slightly higher (69.3%) in PPL liposomes (L-PPL). Similar results were reported in a previous work, where a pomegranate polyphenolic extract was encapsulated in partially purified soy phosphatidylcholine liposomes with an EE of 63% [2]. On the other hand, greater entrapment efficacy (>92%) in rapeseed lecithin liposomes has been reported for apigenin [5]. In this connection, the EE of lactoferrin encapsulated in rapeseed phospholipid liposomes has been shown to vary between 61% and 92% depending on phospholipid concentration, sonication time and the amount of stabilizer added (stigmasterol) [4].

**Figure 3:**
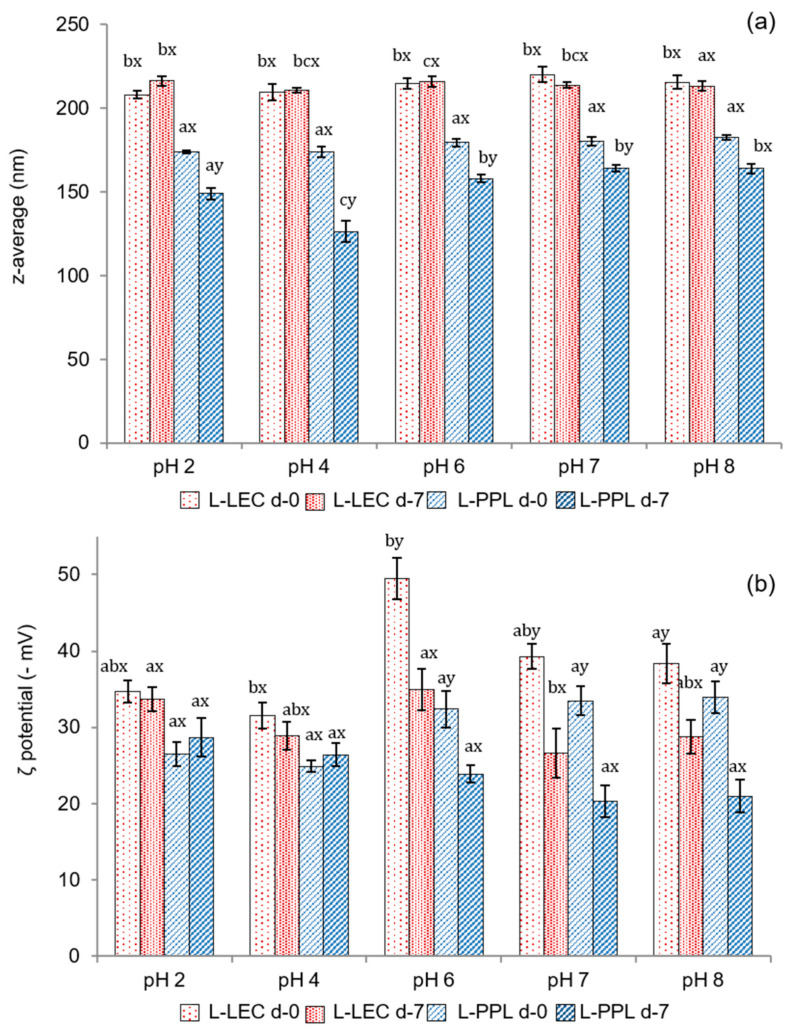
(**a**) Mean particle size (expressed as z-average, nm) and (**b**) ζ potential (−mV) of newly prepared (d-0) liposomal suspensions at different pHs and after 7 days of chilled storage (d-7). L-LEC: liposomes made of rapeseed lecithin loaded with chia extract; L-PPL: liposomes made of partially purified phospholipid fraction loaded with chia extract. Different letters (a,b,c) indicate significant differences (*p* < 0.05) as a function of pH. Different letters (x;y) indicate sig-nificant differences (*p* < 0.05) as a function of storage day.

The pH stability, in terms of particle size and ζ potential, of the freshly prepared liposomal suspensions and after 7 days of storage, is shown in Figure 3. L-LEC liposomes presented z-average values in the range between 220 nm at pH 7 and 208 at pH 2, indicating very little variation as a function of pH (Figure 3a). Furthermore, particle size changed minimally after 7 days, being the sample at pH 2 the only one to exhibit a slight (*p* < 0.05) increase. The L-PPL liposomes were characterized by a smaller particle size (173–182 nm), regardless of pH. In both cases, encapsulation of the chia extract produced a considerable increase in particle size at pH 7, since the corresponding empty liposomes presented z-average of 123 and 102 nm for L-LEC and L-PPL, respectively, at this pH (data not shown). A previous work comparing soy lecithin and partially purified phosphatidylcholine already found that the particle size of liposomes prepared with the latter was smaller [11]. Unloaded rapeseed lecithin liposomes with an average particle size of 125 nm have been reported previously [6]. The particle size of rapeseed phospholipid liposomes encapsulating lactoferrin has been shown to vary from 98 to 204 nm depending on composition and processing parameters [4]. At the different pHs tested in the present study, there was a slight decrease in particle size of L-PPL liposomes (164–126 nm) with storage time, especially at pH 4, denoting slight instability—in principle unrelated to particle fusion or aggregation phenomena. The chia extract did not induce particle reduction in newly prepared liposomes made from either LEC or PPL; however, the decrease of L-PPL average size with time may indicate some chemical instability possibly attributable to interactions with the non-entrapped extract, phospholipid degradation, or occasional leaking from the carrier [35].

Figure 3b presents the ζ potential of the liposomal suspensions at different pHs. The maximum electronegative value in the fresh liposomal suspensions was recorded at pH 6 in L-LEC (−49.5 mV), indicative of very high particle stability. High electronegative ζ potential values have typically been reported for liposomes made from rapeseed and soy lecithin and extracted phospholipids [4,5,11]. There was some slight instability in L-LEC preparations at other pHs tested, but in none of the cases did the ζ potential drop below −30 mV. There was a further surface charge decrease in L-LEC liposomes with storage time, which became significant (*p* < 0.05) only at pH 6–8. The corresponding L-PPL suspensions showed lower ζ potential (in absolute values) than their L-LEC counterparts, with maximum values between −32.4 and −33.9 mV at pH 6–8, and noticeable particle destabilization after 7 days of storage. These findings again indicate lower vesicle stability with the partially purified phospholipid fraction than with the parent rapeseed lecithin. The presence of “impurities” in the LEC sample, especially sterols, might have contributed considerably to the correspondingly higher liposomal membrane stability [36]. In addition, the presumptive concentration of cerebrosides in PPL could have played a role in such relative instability. In this connection, ceramides in phospholipid bilayers have been reported to produce lateral phase separation, inducing membrane permeabilization and occasional transitions to nonlamellar phases [34].

### 3.6. Antioxidant Activity and Phenolic Composition

**Figure 4:**
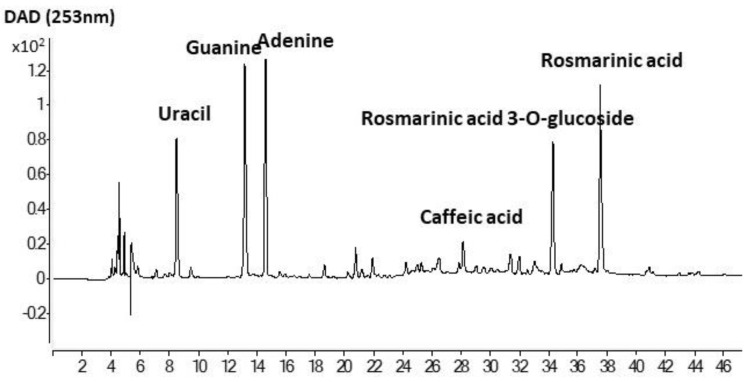
HPLC profile of ethanol/water extract of de-oiled cake of chia seeds.

The Folin-reactive substances (FRS), radical scavenging capacity (ABTS) and ferric ion reducing power (FRAP) of liposomal suspensions (pH 7), and chia extract (ChE) are shown in Table 2. The antioxidant properties of ChE were noticeably higher (*p* < 0.05) than the corresponding liposomal preparations. A similar total phenolic content determined by the Folin–Ciocalteu method was reported by Alcântara et al. [14] for a water/ethanol (33:67) extract from chia flour (46 mg GAE/g), having also a high radical scavenging capacity (DPPH method) and Ferric ion reducing power (FRAP). Rosmarinic acid was the most abundant phenolic compound determined by those authors in that extract, followed by salicylic acid and myricetin. An exhaustive identification of phenolic compounds in chia defatted meal was previously reported by Rahman et al. [37], denoting the presence of a high variety of phenolic acids, flavonoids, and proanthocyanidins. The antioxidant capacity of the chia extract in the present work could be ascribed to the abundance of rosmarinic acid and rosmarinic acid glucoside (Figure 4). In particular, the concentrations of both compounds in ChE were 10.22 ± 0.47 and 6.05 ± 0.24 mg/g, respectively. Other prominent compounds in the present extract were caffeic acid, as well as adenine, guanine, and uracil, indicating that a certain amount of nucleic acids was extracted along with the phenolics, probably favored by the initial mechanical crushing of chia seeds with liquid nitrogen. Nucleic acids are well known to be involved in seeds germination, and an adequate extraction could be useful for authentication in processed food [38]. Table 3 shows other minor phenolic acids that could be identified in this extract by HPLC-MS analysis, such as quinic, caftaric, salvianolic, fertaric, ferulic, ellagic, *p*-hydroxybenzoic, and protocatechuic acids, among others. Pigni et al. [16] recently reported a quite similar composition, with both rosmarinic acid and rosmarinic acid glucoside as the two major components, in an acetone/water extract from partially de-oiled chia flour.

As shown in Table 2, the ABTS and FRAP values were significantly lower (*p* < 0.05) in L-PPL than in L-LEC. This could be attributed in part to the higher EE of the antioxidant chia extract in L-PPL (69.3% in L-PPL vs. 61.3% in L-LEC), which makes the extract less accessible to interaction with the medium. Furthermore, there was a considerable depletion of antioxidant compounds, including sterols and tocopherols, during the acetone-washing step in PPL preparation (Table 1). In this sense, the content of the main sterols determined in LEC decreased by 93% in PPL, while tocopherols decreased by 96%. This effect could also have promoted the poorer antioxidant properties and presumably higher chemical instability of the resulting L-PPL liposomes.

### 3.7. Cytotoxicity

L-LEC liposomes, which registered a higher antioxidant capacity and particle stability, were selected to evaluate cytotoxicity and proinflammatory or anti-inflammatory cytokine production. The biocompatibility of the samples depends on their concentration as well as their biological properties, and therefore, two sample concentrations were used for incubation with Caco-2 and THP-1 cells. For comparative purposes, empty lecithin liposomes (E-L) and chia extract (ChE) were also tested. All samples were found non-cytotoxic, regardless of the concentration tested, with cell viability >90% (data not shown). The low sample concentration was selected for further analysis of immune stimulation.

### 3.8. Anti-Inflammatory Activity

**Figure 5:**
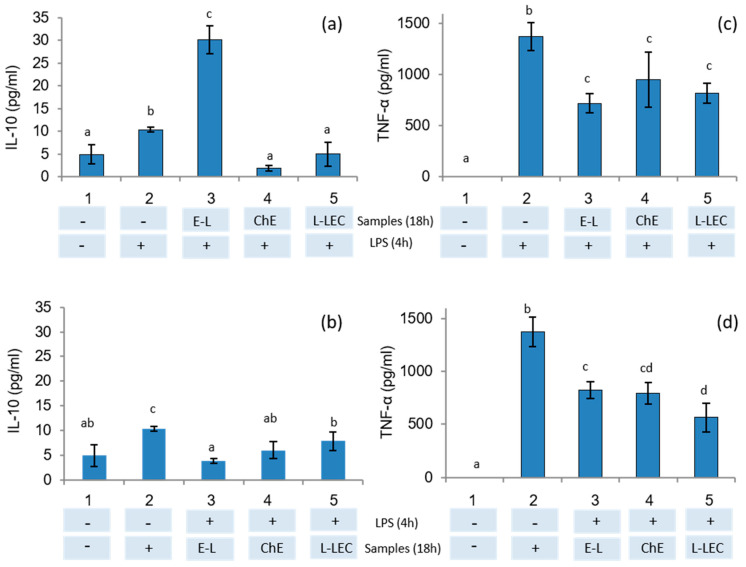
IL-10 and TNF-α secretion in THP-1 cells differentiated into macrophages tested by ELISA kit. (**a**,**c**) Cells were treated with samples E-L (500 μg/mL), chia extract (160 μg/mL), and L-LEC (660 μg/mL) for 18 h and then stimulated with LPS (1 μg/mL) for 4 h. (**b**,**d**) Cells were stimulated first with LPS and then treated with the samples. 1: control cells, 2: control cells treated with LPS, 3 E-L, 4: ChE, and 5: L-LEC. E-L: empty liposomes; ChE: chia extract; and L-LEC: liposomes made of rapeseed lecithin loaded with chia extract. Different letters (a,b,c) indicate significant differences (*p* < 0.05) among samples.

To study the anti-inflammatory effects of E-L, ChE, and L-LEC liposomes, THP-1 monocyte cells were firstly differentiated into macrophages. In a first assay, macrophages were pretreated with samples and then stimulated with LPS to verify their protective anti-inflammatory activity. In a second assay, macrophages were first stimulated with LPS and then treated with the samples to study the ability of these samples to downregulate the inflammation response, as reported previously by De Marco et al. [39]. The results of the present study showed that only the supernatant of the cells incubated with the empty liposomes (E-L) before LPS stimulation induced significant IL-10 secretion (Figure 5a). Then, when macrophages were in the inflammatory state induced by pretreatment with LPS, no induction of IL-10 production was observed in the presence of the three samples tested (Figure 5b). On the other hand, a downregulation of TNF-α production in the supernatants was measured when the cells were pretreated with the three samples and subsequently stimulated with LPS (Figure 5c). Similarly, when macrophages were in an LPS-induced inflammatory state, all samples again showed downregulation of the inflammation response (Figure 5d). THP-1 cells have been widely used as in vitro models of human monocytes and macrophages in mechanistic studies of inflammatory diseases [40]. Moreover, stimulation of PMA-differentiated THP-1 cells with LPS has been proposed as a useful in vitro screening tool to study inflammation-modulating food compounds [41]. Some studies have analyzed the impact of food compounds specifically on the immune system: one of the first was a preliminary report about the impact of chia (*Salvia hispanica*) in rats [42]. Other authors revised the therapeutic perspectives of chia seeds as anti-inflammatory agents as well as other beneficial effects on human health [43]. In the present research, because IL-10 is an anti-inflammatory cytokine that downregulates the proinflammatory cascade, our results indicate that the empty liposomes (E-L) present significant protective anti-inflammatory activity. In addition, this E-L, as well as the ChE and the L-LEC, reduced the production of the proinflammatory cytokine TNF-α caused by stimulation of macrophages with LPS.

## 4. Conclusions

The results of the present study indicate that the mean size and stability of liposomes varied depending on differences in the chemical composition of the parent lecithin and the partially purified phospholipid fraction. The higher sterol and tocopherol concentrations in lecithin endow the resulting liposomes with higher antioxidant activity and particle stability. The chia extract showed noticeable antioxidant properties, associated with a variety of identified phenolic compounds. Both chia extract and lecithin liposomes showed no cytotoxicity to Caco-2 and THP-1 cells and induced downregulation of the inflammation response. The natural origin of these materials represents a sustainable source for producing liposomes that could be useful in the formulation of functional foods. However, more in-depth studies are needed to evaluate the oral bioavailability of these liposomes after in vitro gastrointestinal digestion and by performing in vivo trials.

## Figures and Tables

**Table 1:** Chemical composition of commercial rapeseed lecithin (LEC) and partially purified phospholipid fraction (PPL).

	LEC	PPL
**Fatty Acids (g/100 g)**		
C14:0	0.1 ± 0.0	0.1 ± 0.0
C16:0	7.3 ± 0.0	9.6 ± 0.2
C16:1n7	0.3 ± 0.0	0.4 ± 0.0
C17:0	0.1 ± 0.0	0.1 ± 0.0
C18:0	1.2 ± 0.0	0.9 ± 0.1
C18:1n9c	56.0 ± 0.4	51.8 ± 0.5
C18:1n7c	2.6 ± 0.0	2.3 ± 0.0
C18:2n6c	25.0 ± 0.1	29.9 ± 0.3
C18:3n3	6.0 ± 0.0	4.3 ± 0.0
C20:0	0.3 ± 0.0	0.1 ± 0.0
C20:1n9	0.7 ± 0.0	0.2 ± 0.0
C22:0	0.2 ± 0.0	0.1 ± 0.0
C24:0	0.1 ± 0.0	0.1 ± 0.0
∑ SFA	9.4	11.1
∑ MUFA	59.6	54.7
∑ PUFA	31.0	34.2
**Sterols (mg/100 g)**		
Campesterol	223 ± 23.8	12.81 ± 1.37
Stigmasterol	5.83 ± 0.62	4.07 ± 0.44
β-Sitosterol	215 ± 23.0	15.23 ± 1.63
Cholesterol	2.08 ± 0.22	n.d.
Cycloartenol	n.q.	n.q
Stigmasterol der.	n.q	n.d.
**Tocopherols (mg/100 g)**		
γ-tocopherol	18.1 ± 0.12	0.81 ± 0.18
δ-tocopherol	0.84 ± 0.01	0.04 ± 0.00
α-tocopherol	1.53 ± 0.09	0.03 ± 0.00
**Amino Acids (mg/100 g)**		
Asp	2.70 ± 0.94	3.84 ± 0.07
Thr	0.75 ± 0.30	1.15 ± 0.32
Ser	1.86 ± 0.49	3.15 ± 0.28
Glu	3.68 ± 1.44	3.71 ± 0.21
Gly	1.42 ± 0.52	2.02 ± 0.06
Ala	1.04 ± 0.41	1.40 ± 0.10
Cys	0.46 ± 0.08	0.68 ± 0.05
Val	1.51 ± 0.51	2.23 ± 0.38
Met	0.38 ± 0.34	0.32 ± 0.05
Ile	0.88 ± 0.44	1.13 ± 0.20
Leu	1.52 ± 0.67	1.91 ± 0.29
Tyr	0.86 ± 0.31	0.87 ± 0.21
Phe	0.82 ± 0.29	1.48 ± 0.26
His	0.64 ± 0.29	1.05 ± 0.09
Lys	1.21 ± 0.36	2.06 ± 0.18
Arg	0.63 ± 0.31	0.90 ± 0.10
Pro	1.16 ± 0.36	2.46 ± 1.28

n.q.: not quantified; n.d.: not detected.

**Table 2:** Antioxidant activity (ABTS and FRAP assays) and Folin-reactive substances (FRS) of chia extract (ChE) and liposomes made of commercial rapeseed lecithin (L-LEC) and partially purified phospholipid fraction (L-PPL), both loaded with the chia extract.

Sample	FRS (mg GAE/g)	ABTS (mg Vit C eq./g)	FRAP (mM Fe^2+^ eq./g)
L-LEC	10.7 ± 1.0 ^a^	11.1 ± 0.2 ^a^	179.1 ± 8.2 ^a^
L-PPL	9.5 ± 1.4 ^a^	9.7 ± 0.1 ^b^	143.3 ± 5.0 ^b^
ChE	42.2 ± 3.7 ^b^	26.1 ± 1.3 ^c^	922 ± 69 ^c^

Results are the mean ± standard deviation. Different letters indicate significant differences (*p* < 0.05) among samples.

**Table 3:** Identification of phenolic compounds and others by HPLC/MS.

Tentative Identification	Retention Time (min)	[M-H] (m/z)	Molecular Formula	Score (%)	MS/MS Fragments (m/z)
Malic acid	6.22	133.01	C_4_H_6_O_5_	96	71, 79, 116
Citric acid	8.52	191.02	C_6_H_8_O_7_	95	111, 87
Quinic acid	18.62	191.05	C_7_H_12_O_6_	99	101, 114, 85
Protocatechuic acid-O-hexoside	19.18	315.07	C_13_H_16_O_9_	98	153, 109
Protocatechuic acid	21.12	153.02	C_7_H_6_O_4_	99	109
Salicylic acid glucoside	21.65	299.08	C_13_H_16_O_8_	95	137, 237
p-Hydroxybenzoic acid	24.72	137.02	C_7_H_6_O_3_	91	108, 92
Caffeic acid hexoside	25.13	341.09	C_15_H_18_O_9_	90	135, 179
Caftaric acid	26.23	311.04	C_13_H_12_O_9_	92	135, 179
Caffeic acid	28.20	179.04	C_9_H_8_O_4_	87	135
Salvianolic acid I/H	30.03	537.11	C_27_H_22_O_12_	94	269,183, 109
Coutaric acid	30.90	295.05	C_13_H_12_O_8_	84	179, 133, 71
Fertaric acid	31.49	325.06	C_14_H_14_O_9_	96	193, 134
Rosmarinic acid 3-O-glucoside	34.49	521.13	C_24_H_26_O_13_	98	161, 323, 179
Ferulic acid	35.79	193.05	C_10_H_10_O_4_	97	90
Ellagic acid	36.09	301.00	C_14_H_6_O_8_	97	145, 228
Salvianolic acid E/B/L	36.58	717.15	C_36_H_30_O_16_	89	519, 338, 118
Rosmarinic acid	37.87	359.08	C_18_H_16_O_8_	99	161, 135, 73
Salvianolic acid C	41.12	491.10	C_26_H_20_O_10_	88	311, 267, 123

## Data Availability

Data is contained within the article.
